# Santulli Procedure Revisited in Congenital Intestinal Malformations and Postnatal Intestinal Injuries: Preliminary Report of Experience

**DOI:** 10.3390/children9010084

**Published:** 2022-01-07

**Authors:** Nicolas Vinit, Véronique Rousseau, Aline Broch, Naziha Khen-Dunlop, Taymme Hachem, Olivier Goulet, Sabine Sarnacki, Sylvie Beaudoin

**Affiliations:** 1 Department of Pediatric Surgery and Urology, Necker-Enfants Malades Hospital, APHP, 75015 Paris, France; nicolas.vinit@gmail.com (N.V.); veronique.rousseau@aphp.fr (V.R.); aline.broch@aphp.fr (A.B.); naziha.khen-dunlop@aphp.fr (N.K.-D.); sabine.sarnacki@aphp.fr (S.S.); 2 Faculté de Médecine Paris Centre, Université de Paris, 75006 Paris, France; olivier.goulet@aphp.fr; 3 Department of Neonatology, Necker-Enfants Malades Hospital, APHP, 75015 Paris, France; taymme.hachem@aphp.fr; 4 Department of Gastroenterology, Hepatology and Nutrition, Necker-Enfants Malades Hospital, APHP, 75015 Paris, France

**Keywords:** neonatal surgery, Santulli procedure, intestinal atresia, necrotizing enterocolitis, Hirschsprung’s disease

## Abstract

In our experience, the Santulli procedure (SP) can improve bowel recovery in congenital intestinal malformations, necrotizing enterocolitis (NEC), and bowel perforation. All cases managed at our institution using SP between 2012 and 2017 were included in this study. Forty-one patients underwent SP (median age: 39 (0–335) days, median weight: 2987 (1400–8100) g) for intestinal atresia (51%, two gastroschisis), NEC (29%), midgut volvulus (10%), Hirschsprung’s disease (5%), or bowel perforation (5%), with at least one intestinal suture below the Santulli in 10% of cases. The SP was performed as a primary procedure (57%) or as a double-ileostomy reversal. Anal-stool passing occurred within a median of 9 (2–36) days for 95% of patients, regardless of the diversion level or the underlying disease. All three patients requiring repeated surgery for Santulli dysfunction had presented with stoma prolapse (*p* < 0.01). Stoma closure was performed after a median of 45 (14–270) days allowing efficient transit after a median of 2 (1–6) days. After a median follow-up of 2.9 (0.7–7.2) years, two patients died (cardiopathy and brain hemorrhage), full oral intake had been achieved in 90% of patients, and all survivors had normal bowel movement. Whether used as primary or secondary surgery, the SP allows rapid recovery of intestinal motility and function.

## 1. Introduction

Primary bowel anastomosis or diversion may be considered in congenital intestinal malformations and necrotizing enterocolitis of the preterm neonate [1,2,3,4,5,6,7]. The Santulli procedure (SP) was initially described for the management of intestinal atresia as a promising alternative given the high rate of complications, such as malfunction or leakage, seen in primary anastomosis attempts [8]. Avoiding complete diversion of the distal bowel (small intestine and colon) was highlighted as a means to further enhance intestinal motility and colonic function [9].

**Figure 1:**
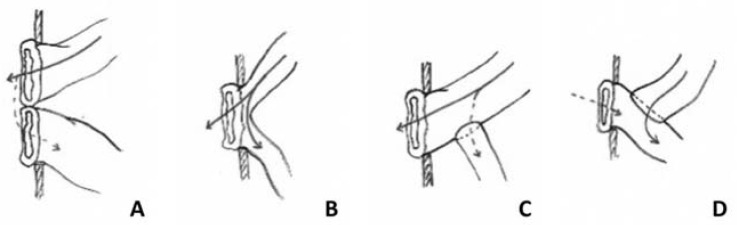
Intestinal stomas: double enterostomy (**A**), lateral enterostomy (**B**), Santulli’s enterostomy (**C**), and Bishop–Koop enterostomy (**D**).

Other procedures have been promoted [6] but the SP, promoted since 1961, offers advantages and deserves to be revisited. Allowing the prompt use of the proximal bowel with trophic enteral feeding was also stressed as a major factor for intestinal growth and maturation [10]. In our clinical practice, we have also observed adverse events in both anastomosis and double enterostomy performed for intestinal malformations and/or enterocolitis. Therefore, convinced that Santulli’s enterostomy (Figure 1) may improve the recovery of the damaged bowel in such cases, we used this technique adapted to various indications and herein report our preliminary experience.

## 2. Materials and Methods

The present study was retrospectively conducted in a tertiary pediatric care center. We searched our institution’s medical database for cases of SP performed between January 2012 and December 2017.

**Figure 2:**
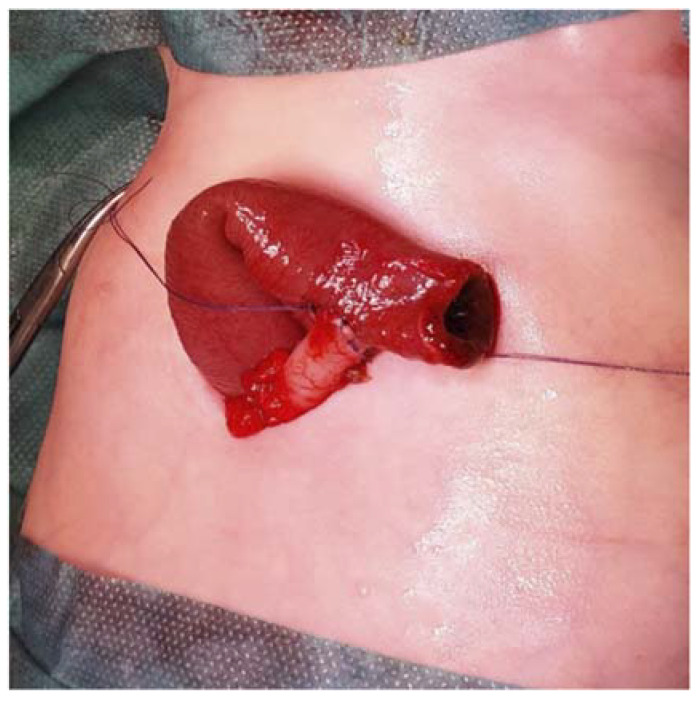
End-to-side anastomosis.

In all cases, an end-to-side intestinal anastomosis was performed with interrupted absorbable suture using braided or monofilament thread, and the proximal segment was brought out to the skin, where it was stitched in the same way (Figure 2). The intestinal length of the diverted proximal part was about 2 to 3 cm, depending on the thickness of the abdominal wall. No catheter was placed within the distal part.

All medical charts were reviewed and clinical data were collected. Postoperative items of interest included age and body weight at SP, mortality (defined as death within the first year), time to first anal stool, age and weight at closure, time to effective transit after Santulli closure, enteral feeding and oral intake after closure, need for lasting enteral support, need for parenteral nutrition, time to discharge, and complications such as stoma prolapse or stricture and need for subsequent surgery. There were no missing data.

The clinical outcomes were then compared according to the underlying condition, the level of diversion, and whether the SP was used as the primary or secondary surgery.

Interquartile range (IQR) and outliers are reported for variables expressed as medians. For statistical analysis, we used the chi-squared and Fisher’s exact tests for numeric variables and the Mann–Whitney test for categorical variables. A *p*-value < 0.05 was considered significant.

## 3. Results

### 3.1. Population

Forty-three patients managed with the SP were identified. Two cases were excluded because the children had been referred solely for the operative time and were otherwise managed in another center. The 41 remaining patients represent our study population (Table 1). The median gestational age was 33 weeks of amenorrhea (WA) (24.3–40.3, IQR = 7) and median birth weight was 2035 g (660–4175, IQR = 1391). There were 24 girls. Half of the patients (21/41) had intestinal atresia, two of which were associated with gastroschisis. Other diagnoses included necrotizing enterocolitis (NEC), not in the acute state, in 29% of cases (12/41); midgut volvulus in 10% of cases (4/41), including one meconium ileus due to cystic fibrosis; Hirschsprung’s disease in 5% (2/41) of cases; and bowel perforation in 5% (2/41) cases.

### 3.2. Indication and Type of Surgery

The SP was performed as primary surgery in 57% of cases (23/41). Among them, 74% (17/23) had intestinal atresia and 26% (6/23) were managed for bowel stenosis after medically managed NEC. In 24% of cases (10/41), this procedure was applied as a means of a double ileostomy reversal, including three children with intestinal atresia, five with a history of NEC, and two with primary intestinal perforation. The choice was made by the surgeon either because of a lasting discrepancy (>4:1) between bowel segments, or when the aspect and/or the trophicity of the distal part of the bowel was not deemed satisfactory enough to perform the anastomosis. Lastly, this procedure was used in 19% of cases (8/41) for the management of major complications (six obstructions and two leakages) of a previous anastomosis. The two children with Hirschsprung’s disease belonged to the latter group, with a rather complicated course. The first child had an ileoanal anastomosis for total colonic aganglionosis with several episodes of enterocolitis and persistent bowel obstruction. At reoperation, the small bowel was found to be atonic with signs of enteritis, thus leading to the decision to use a Santulli diversion. The second child initially presented with enterocolitis with subsequent small bowel volvulus complicated with abdominal compartment syndrome, multiple small bowel resections, and persistent small bowel obstruction. A Santulli enterostomy was thus performed at the time of the Duhamel procedure because a significative size discrepancy was found above ischemic and fibrotic strictures, which were resected, and to protect the colorectal anastomosis. When used as a secondary procedure, the median number of surgeries preceding the Santulli stoma was 1 (1–5, IQR = 2).

The median age at surgery was 37 days (0–335 days, IQR = 90) with a median weight of 2975 g (1400–7600 g, IQR = 1488), with a significantly lower weight (median: 2360 g (1800–3790g, IQR = 683)) in patients with jejunal atresia (*p* = 0.01). Weight at surgery was lower than 2500 g in 14/41 (33%) of cases.

The median length between the duodenojejunal flexure (DJF) and the anastomosis was 61 cm (11–230 cm, IQR = 84). The median length of the distal small bowel segment was 28 cm (0–147 cm, IQR = 67). The distal end consisted solely in the colon in two cases. The anastomosis was located at less than 30 cm from the DJF in 21% (9/41) of cases, all of whom had jejunal atresia and two with the apple-peel type. In 10 cases, the diverted anastomosis was performed above at least another intestinal suture: one patient had a Duhamel procedure, one had an ileoanal anastomosis, and seven had either midgut or colonic resection with end-to-end anastomosis. One of these patients had two intestinal sutures below the Santulli stoma.

### 3.3. Postoperative Course

Death occurred for two patients (5%). Both were preterm neonates with midgut atresia and had been operated on shortly after birth. The first one presented with multiple malformations, including congenital cardiopathy, and died from cardiac failure at day 7. He was, therefore, excluded from the postoperative course results. The other was a twin affected by twin–twin-transfusion syndrome and died at day 54 from severe brain hemorrhage. Although stool had passed through the anus at postoperative day 9, she died before stoma closure.

No leakage of the end-to-side anastomosis or stoma stricture was observed. Stoma prolapse occurred in about 10% of cases (4/41). Two appeared in primary procedures and the other two in secondary procedures.

Onset of stool passing through the anus was noted at a median time of 9 days (2–36 days, IQR = 8) in 38/40 children (excluding the early dead patient). This time was found to be independent of the diversion level (*p* = 0.28) or the underlying disease (*p* = 0.38). In two cases, transit below the stoma was not established after 2 months.

Stoma closure was performed after a median time of 45 days (17–270 days, IQR = 48) in 39/40 patients. No remaining size mismatch between both bowel segments was found, and the distal part of the bowel showed proper trophicity, i.e., normal coloration, wall thickness, and peristaltic movements. In the bowel atresia group, the median stoma duration (39 days, range 21–240 days, IQR = 33) was slightly but nonsignificantly shorter (*p* = 0.43) (Table 2). Two patients, both with a complicated surgical course prior to this procedure, remained with the diversion at the end of the study (one with complex gastroschisis and the other with Hirschsprung’s disease). The median time for effective transit after stoma reversal was 2 days (1–6 days, IQR = 2). No eventration was observed on the site of closure.

Overall, full enteral feeding was achieved in 92% of living cases (36/39) after a median time of 4 days (1–182 days, IQR = 8). Of the 39 surviving patients, 3 (8%) had short-bowel syndrome (SBS) with a small bowel length at SP of 42 cm at 36.3 WA, 38 cm at 41.4 WA, and 125 cm at 6 months. Except for these three patients requiring long-term parenteral nutritional (PN), all children were discharged weaned off PN. Only one patient (with esophageal atresia) who needed a gastrostomy was discharged with a feeding tube. Patients were discharged home after a median time of 14 days (2–126 days, IQR = 21) following stoma closure.

Three children (8%) required subsequent surgical procedures, all for anastomosis dysfunction. All of them had initially presented with stoma prolapse (*p* < 0.01). For one of them, a second SP was necessary after 2 months, with an uneventful further course. Another one (complex gastroschisis) presented with bowel obstruction 4 months after stoma closure and had a second Santulli procedure. The last one needed repeat surgery 2 years after stoma closure because of persistent jejunal dilatation and frequent vomiting. At surgical procedure, however, there was no anastomotic stricture. Jejunal tapering was then performed with satisfactory results.

### 3.4. Follow-Up

The median follow-up was 2.9 years (0.7–7.2 years, IQR = 1.5). At their last visit, all surviving patients with bowel continuity had normal bowel movements. Full oral intake was achieved in 35/39 of living cases (90%).

## 4. Discussion

We herein reported our results of Santulli enterostomy applied in congenital intestinal malformations and postnatal intestinal injuries. This procedure is safe and effective in decompressing obstructed bowels with a low rate of complications and minimal bowel shortening. Final anastomosis can be performed quickly without the need for contrast enema. Hospital discharge delay was also shorter than that reported after other procedures.

Temporary enterostomy is widely used in neonates, either for intestinal malformations such as bowel atresia, or in acquired intestinal affections including NEC and bowel perforation. Although considered an effective salvage procedure, it entails its own complications [11,12,13], including an increased need for PN especially when the distal segment is long [11,14]. Some authors have suggested that primary anastomosis should be preferred to temporary diversion [1,5,15], but others reported a high complication rate, for acute disease such as NEC, isolated intestinal perforation [7,16], and bowel atresia [2]. This prompted Santulli and Blanc to design a novel enterostomy for intestinal atresia [8]. The mirror technique previously described by Bishop and Koop was designed to give access to the distal segment for irrigation with pancreatic extracts in meconium ileus, not to achieve decompression of the proximal segment and stimulation of the lower part [17]. Misused in bowel atresia, when the distal part of the intestine is small and immature, it has a reported complication rate of 41% [18].

The Santulli enterostomy avoids the non-use of the distal bowel, especially the colon, restoring the enterohepatic circulation, preserving intestinal microbiota, avoiding diversion colitis, and so, reducing the risk of cholestasis, sodium depletion, and metabolic acidosis, especially in SBS [19,20]. It prevents any risk of bowel overload. Recent support was provided to such “in continuity” enterostomies to improve bowel function in SBS [21].

In our series, no tube was placed in the distal part, and the proximal content spontaneously forwarded within, as we could observe with feces passing through the anus at a median time of 9 postoperative days. This is the most natural method of continuous irrigation, and it bears no risk of sepsis as the chyme/feces do not stagnate before use [22]. Better than nutrients, the proximal content also brings biliary and pancreatic secretions that will improve the rate of intestinal absorption [23]. Moreover, the use of a feeding tube, as originally described, seems to be an obstructive factor in the small lumen of the distal intestine and might actually counteract the desired purpose.

In this report, 29% of patients were treated for NEC. We did not consider Santulli enterostomy during the acute phase of NEC or intestinal perforation, because it results in the same morbidity as a primary anastomosis [16] and requires additional operating time [4]. In acute NEC, the purpose of intestinal diversion is to achieve full decompression and prevent further bowel ischemia, in order to avoid perforation and progression of the necrosis [7]. Our management in acute NEC is diversion without resection, as described by Luzzatto et al. [24]. In intestinal spontaneous perforation, the massive septic contamination of the abdominal cavity also increases the risk of leakage from any intestinal suture [16]. We performed the Santulli procedure after the acute phase of NEC either to secure the resection of intestinal stenosis or to enhance bowel rehabilitation after a previous enterostomy.

We then used the SP as a first step to continuity restoration in 57% of cases, including intestinal atresia in 70% of patients. This is the initial indication for this “permissive enterostomy” [2,25,26], and we considered it in situations where there was a significant discrepancy (more than 4:1) between proximal and distal segments. When used as a second-step procedure (reversal of a previous double-barrel enterostomy in 24% of cases, management of acquired intestinal stenosis in 15% or management of major complications from a previous anastomosis in 19%), the objective was to allow the progressive restoration of the distal bowel function and to avoid potential complications expected from direct anastomosis. Although SP requires a short additional procedure, no postoperative complication was observed in our cohort, apart from stoma prolapse, compared to the significative rate of complications and additional surgeries of both primary anastomosis and plain enterostomy [2,7,16]. Despite the reported risk of an intraabdominal suture line in patients with direct anastomosis [16], we did not observe any case of anastomosis breakdown or leak in our series. The procedure was performed in low-weight babies without adverse events, which are otherwise more frequent for those under 2660 g, according to Lee et al. [14].

The overall stoma-related complications rate was rather low (4/40) compared to the 40% rate reported for terminal enterostomy in the literature [11,12]. Interestingly there was no stoma stricture or retraction. The complications we observed were stoma prolapses or anastomosis dysfunction or stricture in 10% (4/41) of patients. All patients with Santulli dysfunction had presented with stoma prolapse, and therefore would not benefit from the distal part irrigation. Factors influencing stoma prolapse should be investigated in a larger cohort of patients, but it seemed at reoperation that the chimney length might had been left too long in these cases.

Contrast enema to exclude possible stenosis in the distal segment of the intestine was not necessary since anal stool passing and decreased effluent by the stoma ensured the permeability of the distal segment. Once both criteria were met, stoma closure was performed without additional X-ray exposure, which should be restricted in the pediatric population [27]. Stoma reversal was performed within a median time of 45 days in the whole population. In the intestinal atresia especially, our median time of stoma duration appears strikingly shorter than the 246 days reported by Martynov et al. [28].

Overall, the use of the Santulli procedure, either as a first option for continuity restoration or as a part of a multistep procedure to achieve intestinal continuity, ensured full enteral feeding in 92% of cases (36/39) within a median time of 4 days (1–182 days, IQR = 8) following enterostomy closure. This wide range of time is explained by the need for PN in patients with SBS, even when oral intake was possible. The only patient who needed a gastrostomy had an associated esophageal atresia. Home discharge was achieved after a median time of 14 days (2–126 days, IQR = 21) (for the same reason) following stoma closure. A shorter length of hospital stay was reported by others, mainly with primary anastomosis, but with a much higher complication rate: some authors reported 23% to 25% for the secondary procedure [29,30]. In other series, this rate was as high as 48% [6].

In many cases, despite an appropriate procedure to restore intestinal continuity, digestive autonomy might be delayed because of intestinal dysmotility. For intestinal atresia, evidence linking this dysmotility to anomalies of the enteric nervous system (ENS) in both proximal and distal segments was provided, potentially related to prenatal obstruction [31]. In NEC, it may result from an injury to the ENS and/or the diversion itself [11,16]. Our management of patients with intestinal atresia, NEC-related obstruction, or dysfunctional anastomosis in other intestinal congenital diseases was guided by the conviction that feeding and stimulating the distal segment before anastomosis would have a positive effect on the intestinal wall, mucosal growth, lumen size, and stoma reversal conditions [23]. This segment had been deprived of mechanic and nutritional stimulation since the diversion, at best, and, in some cases, since the prenatal period. Our results showed that the Santulli procedure is safe and effective in neonates to allow a quick recovery after stoma closure. Although it requires an additional procedure compared to direct anastomosis, it prevents episodes of intestinal distension linked to dysmotility, bacterial translocation, or even catheter-related sepsis due to PN dependence.

## 5. Conclusions

Santulli enterostomy can be used as a first-choice surgery any time there is a risk of primary anastomosis, or to manage a complicated former double enterostomy. Our results showed that it allows the rapid recovery of intestinal motility and function after stoma closure, thus supporting the concept that progressive stimulation and irrigation of nutrients and feces into the defunctionalized distal bowel may improve the delay in achieving digestive autonomy in pediatric patients affected by congenital intestinal malformation or NEC.

## Figures and Tables

**Table 1:** Demographics of the study population.

Parameter	Study Population (*n* = 41)	Midgut Atresia (*n* = 21)
Median GA at birth (WA) (IQR)	33 (7) [24.3–40.3]	35 (5) [29.5–40]
Median birth weight (g) (IQR)	2035 (1391) [660–4175]	2360 (806) [1285–4175]
Indication for SP		
NEC	12/41 (29%)	0
Intestinal atresia	21/41 (51%)	21/21 (100%)
Midgut volvulus	4/41 (10%)	0
Hirschsprung’s disease	2/41 (5%)	0
Bowel perforation	2/41 (5%)	0
Median age at SP (days) (IQR)	37 (90) [0–335]	1 (5) [0–180]
Median weight at SP (days) (IQR)	2975 (1488) [1400–7600]	2455 (1126) [1400–7600]
Median DJF–Santulli distance (cm) (IQR)	61 (84) [11–230]	44 (87) [11–230]
Median Santulli–ICV distance (cm) (IQR)	28 (67) [0–147]	65 (95) [0–147]
SP as primary surgery	23/41 (56%)	17/21 (81%)
Median number of surgeries prior to SP	1.4 [0–5]	0.7 [0–3]

GA: gestational age, WA: weeks of amenorrhea, IQR: interquartile range, SP: Santulli procedure, NEC: necrotizing enterocolitis, DJF: duodenojejunal flexure, ICV: ileocecal valve. Data are expressed as medians with (IQR) and [outliers].

**Table 2:** Postoperative data.

Parameter	Study Population (*n* = 41)	Midgut Atresia (*n* = 21)
Stoma complication		
Stoma prolapse	4/41 (10%)	2/21 (10%)
Stoma stricture	0	0
Median time to first anal stool (days) (IQR)	9 (8) [2–36]	11 (9) [4–30]
Median time to stoma closure after SP (days) (IQR) *	45 (48) [17–270]	39 (33) [21–240]
Median age at stoma closure (days) (IQR) *	81 (76) [25–540]	43 (36) [25–420]
Median weight at stoma closure (g) (IQR) *	4010 (1389) [2500–10700]	3540 (990) [2500–5140]
Median time to effective transit after stoma closure (days) (IQR) *	2 (2) [1–6]	2 (2) [1–6]
Median time to hospital discharge after stoma closure (days) (IQR) *	14 (21) [2–126]	13 (25) [2–126]
Median time to full enteral feeding (days) (IQR) *	4 (8) [1–182]	4 (3) [1–40]
Median time to full oral intake (days) (IQR) *	4 (10) [1–556]	4 (27) [1–556]
Need for nutritional support *		
Tube feeding dependence	1/39 (3%)	1/19 (5%)
PN dependence	3/39 (8%)	1/19 (5%)
Need for subsequent surgery *	3/39 (8%)	2/19 (11%)
Median hospital stay following SP (days) (IQR) *	53 (41) [11–326]	52 (33) [11–216]
Survival	39/41 (94%)	19/21 (90%)
Median follow-up (years) (IQR) *	2.9 (1.5) [0.7–7.2]	3.1 (3.5) [1.0–7.2]

IQR: interquartile range, PN: parenteral nutrition. * Excluding the two patients who died. Data are expressed as medians with (IQR) and [outliers].

## Data Availability

The data presented in this study are available on request from the corresponding author. The data are not publicly available due to ethical restrictions.
